# Rolipram Prevents the Formation of Abdominal Aortic Aneurysm (AAA) in Mice: PDE4B as a Target in AAA

**DOI:** 10.3390/antiox10030460

**Published:** 2021-03-16

**Authors:** Saray Varona, Lídia Puertas, María Galán, Mar Orriols, Laia Cañes, Silvia Aguiló, Mercedes Camacho, Marc Sirvent, Vicente Andrés, José Martínez-González, Cristina Rodríguez

**Affiliations:** 1 Departmento de Patología Experimental, Instituto de Investigaciones Biomédicas de Barcelona-Consejo Superior de Investigaciones Científicas (IIBB-CSIC), 08036 Barcelona, Spain; saray.var@gmail.com (S.V.); marorriols@gmail.com (M.O.); 2 CIBER de Enfermedades Cardiovasculares, ISCIII, 28029 Madrid, Spain; lpuertas@santpau.cat (L.P.); mgalana@santpau.cat (M.G.); laia.canes@iibb.csic.es (L.C.); mcamacho@santpau.cat (M.C.); msirvent10@gmail.com (M.S.); vandres@cnic.es (V.A.); 3 Regulatory Mechanisms of Cardiovascular Remodelling Group, Instituto de Investigación Biomédica Sant Pau, 08041 Barcelona, Spain; saguilo@santpau.cat; 4 Regulatory Mechanisms of Cardiovascular Remodelling Group, Institut de Recerca Hospital de la Santa Creu i Sant Pau (IRHSCSP), 08025 Barcelona, Spain; 5 Angiology and Vascular Surgery Service, Hospital Universitari Germans Trias i Pujol, 08916 Badalona, Spain; 6 Vascular Pathophysiology Area, Centro Nacional de Investigaciones Cardiovasculares Carlos III (CNIC), 28029 Madrid, Spain

**Keywords:** abdominal aortic aneurysm, reactive oxygen species, PDE4B, rolipram

## Abstract

Abdominal aortic aneurysm (AAA) is a common life-threatening condition characterized by exacerbated inflammation and the generation of reactive oxygen species. Pharmacological treatments to slow AAA progression or to prevent its rupture remain a challenge. Targeting phosphodiesterase 4 (PDE4) has been verified as an effective therapeutic strategy for an array of inflammatory conditions; however, no studies have assessed yet PDE4 in AAA. Here, we used angiotensin II (AngII)-infused apolipoprotein E deficient mice to study the involvement of the PDE4 subfamily in aneurysmal disease. PDE4B but not PDE4D was upregulated in inflammatory cells from both experimental and human AAA. The administration of the PDE4 selective inhibitor rolipram (3 mg/kg/day) to AngII-challenged mice (1000 ng/kg bodyweight/min) protected against AAA formation, limiting the progressive increase in the aortic diameter without affecting the blood pressure. The drug strongly attenuated the rise in vascular oxidative stress (superoxide anion) induced by AngII, and decreased the expression of inflammatory markers, as well as the recruitment of macrophages (MAC3+), lymphocytes (CD3+), and neutrophils (ELANE+) into the vessel wall. Rolipram also normalized the vascular MMP2 expression and MMP activity, preserving the elastin integrity and improving the vascular remodelling. These results point to PDE4B as a new therapeutic target for AAA.

## 1. Introduction

Abdominal aortic aneurysm (AAA) is a common life-threatening degenerative condition characterized by the progressive weakening and dilation of the arterial wall, which could ultimately lead to vascular rupture. AAA has a prevalence of 4–7% in men over the age of 65 years, while aortic rupture, the more severe complication of this disease, results in extensive internal bleeding, and constitutes a major cause of sudden death among elderly males [1,2,3]. Nowadays, the surveillance of small aneurysms and elective AAA repair is the unique therapeutic approach for AAA, while the finding of effective pharmacological tools that limit the expansion and rupture of the aneurysms remains elusive [4,5]. The development of nonsurgical therapeutic interventions is hampered by the complexity of AAA pathogenesis, which remains only partially characterized.

Chronic vascular inflammation, the proteolytic degradation of the extracellular matrix (ECM), neovascularisation, microcalcification, and vascular smooth muscle cell (VSMC) death by apoptosis are prominent features of AAA [6,7,8,9]. Among them, inflammation is critical in the onset and progression of this disease. Macrophages, lymphocytes, neutrophils, mast cells, and natural killer cells recruited into adventitia and media lead to an exacerbated production of proinflammatory cytokines and chemokines, reactive oxygen species (ROS), and metalloproteinases (MMPs), which are responsible for the proteolytic degradation of ECM [10,11,12].

Cyclic nucleotide phosphodiesterases (PDEs) encompass a vast family of isozymes, which catalyse the hydrolysis and inactivation of cyclic adenosine monophosphate (cAMP) and cyclic guanosine monophosphate (cGMP), second messengers which play a major role in cardiovascular function [13,14,15]. In particular, by controlling cAMP degradation rates, four PDE subfamilies (PDE3, PDE4, PDE7 and PDE8) might impact the vascular function modulating endothelial permeability, monocyte/macrophage activation, and the proliferation, migration, differentiation and contraction–relaxation of VSMC [15,16,17,18]. The PDE4 subfamily, one of the best characterized subfamilies, is constituted by four members (PDE4A-D) with multiple isoform variants expressed in a wide array of tissues and involved in numerous pathophysiological processes. Targeting PDE4 has been validated as an effective therapeutic strategy for inflammatory conditions, including chronic obstructive pulmonary disease (COPD), inflammatory bowel diseases (IBD), rheumatic arthritis (RA), atopic dermatitis (AD), asthma, psoriasis, and lupus [14,15]. PDE4 enzymes seem to play non-redundant functions, but both PDE4D and PDE4B have been involved in inflammation and oxidative stress [19,20,21], and PDE4 inhibitors have been shown to be able to reduce inflammation and oxidative stress both in vitro and in animal models of tissue damage [21,22,23]. Although inflammation and oxidative stress are key hallmarks of AAA, it is uncertain whether PDE4 isoforms could contribute to this human disease, thereby constituting potential targets for pharmacological interventions. Here, we demonstrate that PDE4B expression is upregulated in both human and experimental AAA, and that the inhibition of PDE4 activity reduces inflammation and oxidative stress, preventing the formation of AAA induced by angiotensin II (AngII) infusion in apolipoprotein E deficient (ApoE^−/−^) mice.

## 2. Materials and Methods

### 2.1. Human Samples

Human aneurysmal samples were obtained from patients undergoing the open surgery repair of AAA at the Hospital de la Santa Creu i Sant Pau (HSCSP; Barcelona, Spain). Healthy aortas were collected from multi-organ donors, as previously described [24,25]. Abdominal aorta segments were collected from patients and control donors, following strict standard operating procedures and ethical guidelines. The samples of the control subjects had no post-mortem evidence of AAA, atherosclerotic plaques, or other medical conditions that affect the study. Upon collection, the samples were rapidly stored at −80 °C for subsequent RNA extraction, or were processed for immunohistochemical analysis.

### 2.2. Animal Handling

The animals were housed at the Animal Experimentation Unit (Institut de Recerca HSCSP, Barcelona, Spain) under a standard 12-h light/12-h dark cycle, in a controlled temperature and humidity environment, with ad libitum access to food and water. AAA was induced in 12-week old male apolipoprotein-E-deficient mice (ApoE^−/−^; B6.129P2-Apoe^tm1Unc/J^) by a subcutaneous infusion of AngII, as previously described [26,27]. AngII (1000 ng/kg bodyweight (BW)/min; Sigma-Aldrich, St Louis, MO, USA) was infused via osmotic micropumps (model 1004, Alzet; Durect Corporation, Cupertino, CA, USA) for 28 days [27,28,29]. In brief, the mice were anesthetized with 2% inhaled isoflurane. The anaesthetic depth was confirmed by the loss of blink reflex and/or the lack of response to foot reflex. Then, micropumps were implanted subcutaneously into the interscapular space. The recovery after surgery was carried out using aseptic techniques in a dedicated and approved surgical area. The animals were kept warm in a heating pad until they woke after surgery, and were observed carefully by the investigators throughout the post-surgery period. Analgesics were given immediately after surgery in order to prevent discomfort (buprenorphine, subcutaneously administered once a day during two days; 0.05 mg/kg).

The animals were randomly distributed in three experimental groups: AngII-infused mice; AngII-infused mice receiving a daily intraperitoneal injection of the PDE4 inhibitor rolipram, starting 24 h before the micropump implantation (3 mg/kg; SelleckChem, Houston, TX, USA) [30]; and saline-infused mice, used as controls. At the end of the experimental procedures, the mice were deeply anesthetized via intraperitoneal injection with ketamine (150 mg/kg) and medetomidine (1 mg/kg), sacrificed by thoracotomy, and the aortas were harvested, explored to determine the presence and severity of aneurysms, and appropriately processed for further analysis.

### 2.3. Non-Invasive Measurement of Systolic Blood Pressure

The measurement of systolic blood pressure (SBP) was performed non-invasively in conscious mice prior to and throughout the experimental period using the tail-cuff plethysmography method (CODA^®^ tail-cuff blood pressure system; Kent Scientific Corporation; Torrington, CT, USA). The mice were previously trained for tail-cuff measurements over a period of 1 week. The measurements were recorded every week at the same time (between 9 a.m. and 11 a.m.) in order to avoid the influence of the circadian cycle, and the blood pressure values were derived from an average of at least 6 measurements per animal each day [27,28,29].

### 2.4. Ultrasound Measurement of the Abdominal Aortic Diameter

The abdominal aortic diameter was measured by ultrasound imaging before AngII infusion, and weekly until the end of the experimental protocol. In brief, the mice were anesthetized with inhaled isoflurane (2%) and laid supine onto a warming platform. Then, the precordium was shaved, and abdominal echography was carried out using a Vevo 2100 ultrasound with a 30 MHz transducer applied to the abdominal wall in order to record the abdominal aorta (VisualSonics, Toronto, ON, Canada), as previously described [24,27]. The external diameters of the abdominal aorta with values >1.5 mm were considered as aneurysm. All of the primary measurements were made from images captured on cine loops of 100 frames at the time of the study, using the software provided by the ultrasound machine. The severity of the aneurysm was established as previously described [31], based on a 4-point grading scale: type 0, no aneurysm; type I, dilated lumen in the suprarenal region of the aorta with no thrombus; type II, remodelled tissue in the suprarenal region that frequently contained thrombus; type III, a pronounced bulbous form of type II that contained thrombus; and type IV, a form in which there are multiple AAAs containing thrombus.

### 2.5. Basic Measurements of Cardiac Function by Echocardiography (M-mode and Doppler)

The anesthetized mice (2% isoflurane inhalation) were subjected to a transthoracic echocardiography, using a Vevo 2100 ultrasound with a 30 MHz transducer (VisualSonics, Toronto, Canada) [28,29]. Two-dimensional and M-mode images were obtained in parasternal long-axis and short-axis views, respectively. The following parameters were recorded: heart rate (HR), end-diastolic interventricular septum thickness (IVSTd), left ventricular (LV) end-diastolic volume (LVEDV), LV end-systolic volume (LVESV), LV end-diastolic posterior wall thickness (PWTd), and LV anterior wall thickness at the diastole (AWTd). The LV mass, LV ejection fraction (LVEF) and LV fractional shortening (LVFS) were determined. The LV stroke volume and cardiac output (CO) were calculated according to the following:

LV stroke volume (µL) = LV end-diastolic volume − LV end-systolic volume

CO (mL/min) = (LV stroke volume × HR)/1000

### 2.6. Real-Time Polymerase Chain Reaction (PCR)

The total RNA was isolated from both the mouse and human aortas using TriPure Isolation Reagent (Roche Diagnostics, Mannheim, GE). The RNA (500 ng) was reverse transcribed into cDNA using a High Capacity cDNA Reverse Transcription Kit (Applied Biosystems, Foster City, CA, USA). The quantification of the mRNA levels was carried out by real-time PCR using an ABI PRISM 7900HT sequence detection system (Applied Biosystems). Specific primers and probes (provided by the Assay-on-Demand system; Applied Biosystems) were used for the quantification of the human mRNA levels of *PDE4B* (Hs00277080_m1), *PDE4D* (Hs01579625_m1), and the reference gene, *ACTB* (ß-actin; HS99999903_m1). In order to analyse the mRNA levels in the mouse aorta, the following primers and probes (provided by Applied Biosystems or Integrated DNA Technologies, Coralville, IA, USA) were used: *Mcp1* (Mm.PT.58.42151692), Col1a1 (Mm.PT.58.7562513), *Emr1* (Mm.PT.58.11087779), *Mmp2* (Mm.PT.58.9606100) and Mmp9 (Mm.PT.58.10100097). TATA-binding protein (*Tbp*; Mm00446973_m1) was used as endogenous control for the mouse tissues. The relative mRNA levels were determined using the 2−ΔΔCt method [32].

### 2.7. Western Blotting

The protein lysates were resolved using sodium dodecyl sulphate-polyacrilamide gel electrophoresis (SDS-PAGE) and transferred to polyvinylidene difluoride membranes (Immobilon, Merck-Millipore; Burlington MA, USA, IPVH00010). The blots were blocked with 5% non-fat dry milk in Tris-buffered saline, 0.05% Tween 20 (TBST) buffer at room temperature for 1 h. The membranes were then incubated overnight with antibodies directed against PDE4B (NBP2-01171, Novus Biologicals, Minneapolis, MN, USA), PDE4D (ab171750, Abcam, Cambridge, UK) and ß-actin (A5441, Sigma-Aldrich). The appropriate horseradish peroxidase-conjugated secondary antibodies (Dako Products, Agilent, Santa Clara, CA, USA) and the Luminata^TM^ Western horseradish peroxidase (HRP) Substrate (Immobilon, Merck-Millipore) were used to detect the bound antibodies. The size of the detected proteins was estimated using protein molecular-mass standards (Hyperpage Prestained Protein Marker; Bioline, Paris, France). ß-actin was used to verify the equal loading of the protein on each lane.

### 2.8. Histological and Immunohistochemical Analysis

The arteries were fixed in 4% paraformaldehyde/0.1 M PBS (pH 7.4) for 24 h, and were embedded in paraffin. The tissue sections (5 µm-thick) were deparaffinised in xylene, rehydrated in graded ethanol solutions, and treated with 3% hydrogen peroxide in methanol for 30 min in order to block endogenous peroxidase. The slides were incubated with 10% normal serum, and endogenous avidin and biotin were blocked using a commercial kit (Vector Laboratories, Burlingame, CA, USA). Then, the samples were incubated overnight at 4 °C with the following primary antibodies: MAC3 (sc-19991, Santa Cruz Biotechnology, Dallas, TX, USA), CD3 (A0452, Dako, Agilent Technologies), neutrophil elastase (M0752, Dako, Agilent Technologies), MCP1 (sc-1785, Santa Cruz Biotechnology), or PDE4B (NBP2-01171, Novus Biologicals). The samples were washed and then incubated with the appropriate biotinylated secondary antibodies for 1 h (Vector Laboratories,). After washing three times in PBS, the slides were incubated in avidin-biotin peroxidase complex (Vector Laboratories) for 30 min. The colour was developed by incubation with 3,3′-diaminobenzidine (DAB), and then the sections were counterstained with haematoxylin, dehydrated, cleared, and mounted. The samples in which the primary antibody was omitted were used as the negative controls. The histological characterization of the aortic samples was carried out by Masson trichrome staining, while the elastic fibre integrity was assessed by orcein staining (Casa Álvarez, Madrid, Spain) [27].

### 2.9. In Situ Zymography

The gelatinolytic activity was assessed in optimal cutting temperature compound (OCT)-embedded unfixed frozen tissue sections (8 µm-thick) using Quenched Fluorogenic dye-quenched (DQ)^TM^ gelatin (D-12054, Invitrogen, Carlsband, CA, USA) as a fluorogenic substrate, as previously described [27]. Briefly, DQ-gelatin was dissolved in water at 1 mg/mL and then diluted 1:10 in 1% (*w/v*) low gelling temperature agarose (A9414, Sigma-Aldrich). The mixture was applied onto each section and cover-slipped. The samples were incubated at 4 °C for 30 min in order to allow gelatin gelling, and were then maintained at room temperature for 24 h, protected from light. Fluorescein isothiocyanate (FITC) fluorescence was visualized using Leica TCS SP5 confocal microscopy (excitation wavelength: 495 nm; emission wavelength: 515 nm; 10 × 0.4 DRY UV objective for quantification and 40 × 0.4 OIL UV objective for illustration) and the Leica LAS AF Lite software (Leica Microsystems S.L.U, Barcelona, Spain). Z-stack images using four planes of the same region were captured (1024 × 1024; z-interval, 2.1 µm), and were stacked into a single image using a projection of intensity sum to quantify the fluorescence. The image processing and quantitative fluorescence analysis were performed using FIJI ImageJ software. Negative controls in which the samples were pre-incubated with 20 mM ethylenediaminetetraacetic acid (EDTA) before the addition of the labelled substrate were included [27].

### 2.10. In Situ Detection of Vascular O_2_^·−^ Production

The oxidative fluorescent dye dihydroethidium (DHE, Sigma-Aldrich) was used to detect the in situ production of superoxide anions in the aortic segments [33]. In brief, unfixed aortic segments were embedded in frozen OCT and cut into 8 µm-thick sections using a cryostat microtome. Arterial segments were equilibrated for 30 min at 37 °C in Krebs-HEPES buffer, and then fresh buffer containing 2 µM DHE was topically applied onto each slide, capped with a coverslip, and incubated for 40 min in a light-protected humidified chamber at 37 °C. The fluorescence was visualized using Leica TCS SP5 confocal microscopy (10.0 × 0.40 DRY UV objective for quantification, and 40 × 0.4 OIL UV objective for illustration) and the Leica LAS AF Lite software (Leica Microsystems S.L.U, Barcelona, Spain). The fluorescent images were acquired in a sequential mode, using a 561nm laser (long-wavelength excitation) to detect DHE and a 405 nm (with short-wavelength excitation) laser to detect the autofluorescence of the aorta layers. Z-stack images of four optical sections in the same plane were captured (1024 × 1024; z-interval, 2.1 µm), and were stacked into a single image using a projection of intensity sum to quantify the fluorescence. The image processing and quantitative fluorescence analysis were performed using FIJI ImageJ software (Wayne Rasband, National Institutes of Health, Bethesda, MD, USA) [33].

### 2.11. Statistical Analysis

The results are shown as the mean ± the standard error of the mean (SEM), or as boxplots. In this latter case, the upper and lower boundaries of each box display the 75th and 25th percentiles of data, respectively; the median is indicated by a horizontal line, and the minimum and maximum values are indicated as whiskers. The significant differences were analysed using one way ANOVA, two-way ANOVA with repeated measures, or two-way ANOVA followed by Bonferroni’s post-hoc tests. When the distribution of the data failed the D’Agostino-Pearson omnibus normality test, the Mann-Whitney U test with Tukey’s post-hoc test, or the Kruskal-Wallis test with Dunn’s multiple comparison post-hoc test were applied. The differences in the AAA incidence were analysed by the χ2 test. The data were analysed with GraphPad Prism version 6.01 (GraphPad Software, San Diego, USA). The differences were considered significant at *p* < 0.05.

## 3. Results

### 3.1. Vascular PDE4B Expression Is Enhanced in Mouse and Human AAA

**Figure 1:**
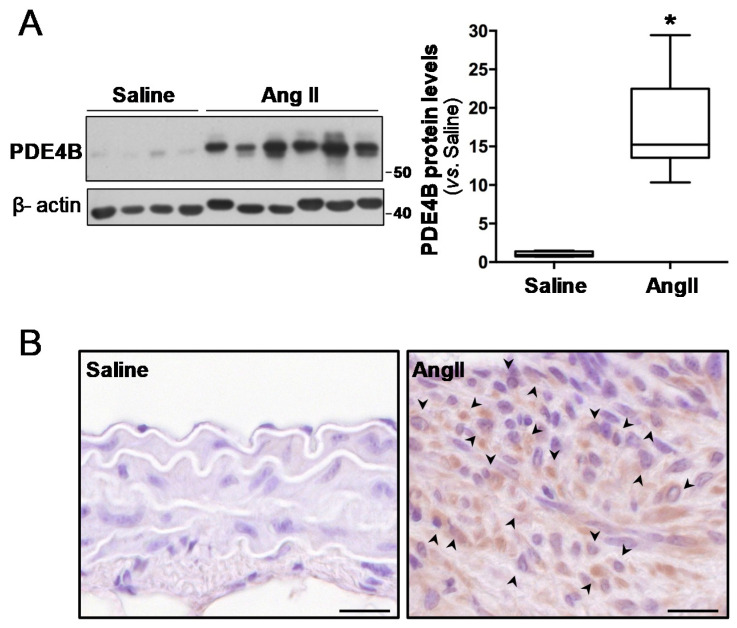
PDE4B expression is up-regulated in the abdominal aortas from AngII-infused ApoE^−/−^ mice. ApoE^−/−^ mice were infused with AngII (1000 ng/kg/min) or saline solution for 28 days. (**A**) The PDE4B expression in abdominal aortas from these animals was assessed by Western blot. The levels of β-actin are shown as a loading control. The boxplot on the right shows the quantification of the PDE4B protein levels. The box extends from the 25th to the 75th percentile, and the median is indicated by a horizontal line. The whiskers represent the maximum and minimum values (Saline, *n* = 4; Ang II *n* = 6); * *p* < 0.01 vs. saline. (**B**) Representative PDE4B immunostaining in these samples. The arrowheads indicate the PD4B-positive cells in aneurysmal tissues. Bars: 20 µm.

Because PDE4B and PDE4D play a major role in inflammation and oxidative stress [34], we analysed the expression of these PDE4 isoenzymes in experimental AAA induced by AngII infusion in ApoE^−/−^ mice, a preclinical model which recapitulates key aspects of the human disease [26]. PDE4B was significantly expressed in mouse AAA (Figure 1). Using an antibody that recognizes the conserved C termini of PDE4B, a major band of 64 kDa significantly up-regulated in AAA was detected by Western blot (Figure 1A and Figure S1). Immunostaining located the PD4B in inflammatory cells infiltrated in aneurysmal tissues (Figure 1B). By contrast, the levels of PDE4D in the healthy aorta and AAA were similar (Figure S2). These data suggest that PDE4B could play a role in AAA development.

**Figure 2:**
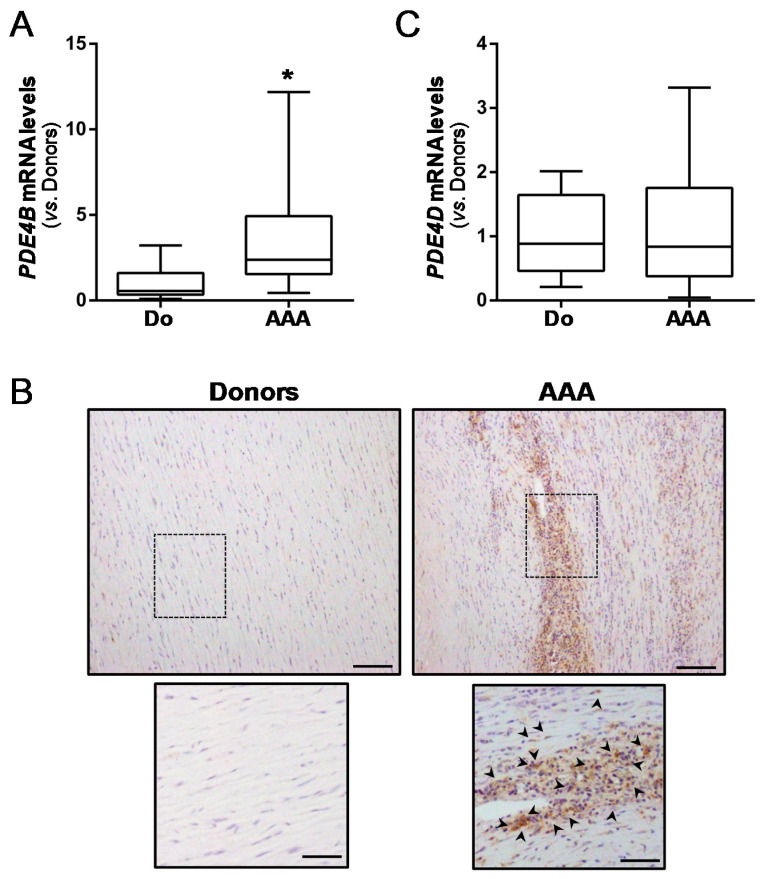
PDE4B expression is enhanced in human AAA. (**A**) PDE4B expression in abdominal aorta from AAA patients (AAA) and healthy donors (Do), assessed by real-time PCR. The data are presented as boxplots. The box extends from the 25th to the 75th percentile, and the median is indicated by a horizontal line. The whiskers represent the maximum and minimum values (DO, *n* = 14; AAA *n* = 61); * *p* < 0.001 vs. Donors. (**B**) Representative PDE4B immunostaining in healthy aorta from donors and aneurysmal tissues from AAA patients (top panels; bar: 100 µm). The boxed areas are shown at a higher magnification below, and the arrowheads indicate PDE4B-positive cells (lower panels; bar: 50 µm). (**C**) PDE4D mRNA levels in these samples.

These results prompted us to address whether PDE4B expression could be similarly regulated in human AAA. Aneurysmal samples from AAA patients undergoing open aortic repair and non-diseased abdominal aortas from organ donors were processed. The clinical and demographic data of the patients and donors are shown in Table S1. Interestingly, in agreement with the data from experimental AAA, the aneurysmal aortas expressed higher levels of PDE4B (≈3.4-fold) than the healthy vessels (Figure 2A), mainly in inflammatory cells (Figure 2B). Conversely, the PDE4D expression remained unchanged (Figure 2C).

### 3.2. Treatment of ApoE^−/−^ Mice with Rolipram Prevents AAA Formation Induced by AngII Infusion

**Figure 3:**
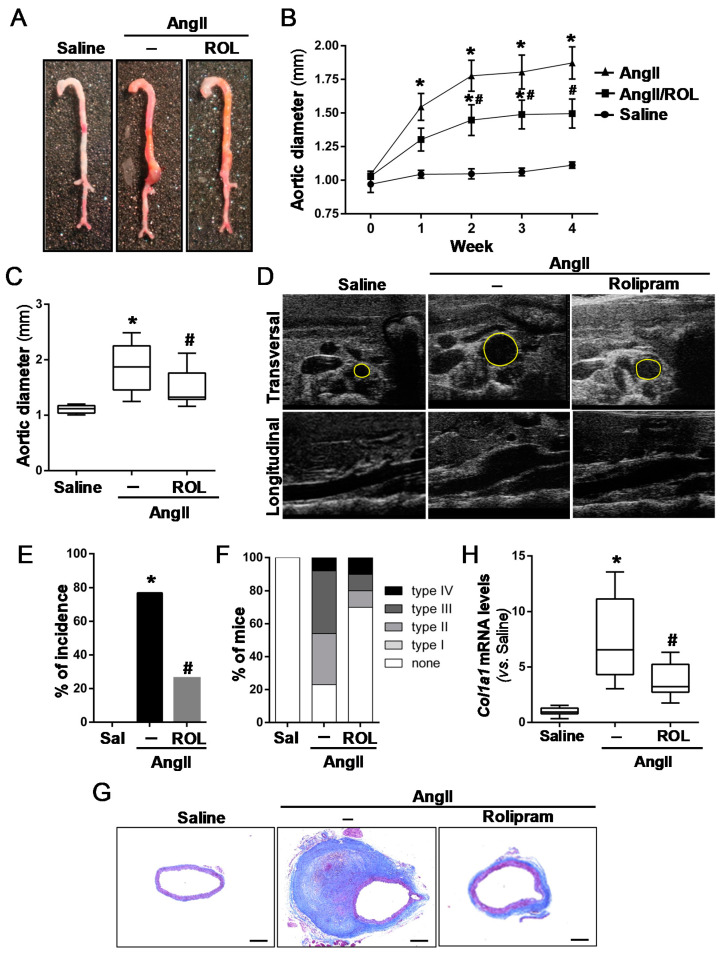
Rolipram ameliorates AAA development in AngII-infused ApoE^−/−^ mice. ApoE^−/−^ mice were infused with saline solution (Sal) or AngII (1000 ng/kg/min) for 28 days. The mice challenged with AngII were treated or not with rolipram (ROL, 3 mg/kg/day). (**A**) Representative macroscopic images of the aortas from each experimental group. (**B**) Time-course analysis of the abdominal aortic diameter by ecochardiography. The data are mean ± SEM (saline-infused mice, *n* = 9; AngII-infused animals, *n* = 13; AngII-infused mice treated with rolipram, *n* = 10). *p* < 0.05: * vs. saline-infused mice; # vs. AngII-challenged mice. (**C**) Quantitative analysis of the aortic diameter at the end of the experimental period (28 days). The results are expressed as boxplots. The box extends from the 25th to the 75th percentile, and the median is indicated by a horizontal line. The whiskers represent the maximum and minimum values. *p* < 0.05: * vs. saline-infused mice at the same time; # vs. AngII-challenged mice. (**D**) Representative ultrasonographic frames after 28 days of AngII infusion. Transverse and longitudinal images taken at the level of the suprarenal aorta are shown. The aortic diameter is indicated with a yellow line. (**E**) Incidence of AAA in each experimental group. *p* < 0.05: * vs. saline-infused mice; #vs. AngII-challenged mice. (**F**) The aneurysm severity based on the Manning scale. (**G**) Masson trichrome staining of the abdominal aortas from each group. Representative images are shown. Bars: 200 µm. (**H**) The Col1A1 mRNA levels assessed in these samples by real-time PCR. The data are presented as box and whisker plots. The box extends from the 25th to the 75th percentile, and the median is indicated by a horizontal line. The whiskers represent the maximum and minimum values (saline-infused mice *n* = 8; AngII-challenged mice, *n* = 12; AngII-challenged mice treated with rolipram, *n* = 8). *p* < 0.05; * vs. saline-infused mice; # vs. AngII-challenged mice.

We sought to determine whether the up-regulation of PDE4B might play an active role in AAA pathophysiology. For this purpose, rolipram, a PDE4 selective inhibitor, was administered to AngII-infused ApoE^−/−^ mice, and their aneurysm progression was monitored by ultrasonography throughout the experimental period. Interestingly, rolipram protected against AAA formation in this model (Figure 3). The macroscopic visualization of isolated aortas at the end of the experimental procedure showed that the abdominal aortic dilation induced by AngII was ameliorated by rolipram (Figure 3A). The weekly ultrasound monitoring of the suprarenal aortic diameter, which estimates the timeline of aneurysm development, revealed an early trend towards a lower vascular dilation in rolipram-treated mice, which was statistically significant after 2 weeks of AngII infusion (Figure 3B–D). This effect was associated with a significant reduction of aneurysm incidence. While 77% of AngII-infused mice developed aneurysms, this percentage decreased to 30% after rolipram treatment (*p* < 0.02) (Figure 3E). Likewise, less severe forms of AngII-induced aneurysm were developed in the mice receiving rolipram (Figure 3F). Masson trichrome staining evidenced the striking disturbance of the vascular morphology exhibited by AngII-challenged mice, and how rolipram improved the vascular phenotype (Figure 3G). Furthermore, this drug attenuated the compensatory increase in aortic collagen I expression induced by AngII (Figure 3H). This drug affected neither the enhanced blood pressure levels (Figure S3) or cardiac hypertrophy (and the compensatory increase of systolic function) evoked by AngII (Table S2), excluding any influence of hemodynamic or cardiac effects on the observed responses. Likewise, bodyweight was not significantly modified by PDE4 inhibition (Table S3).

### 3.3. Rolipram Ameliorates AngII-Induced Inflammation and Oxidative Stress

**Figure 4:**
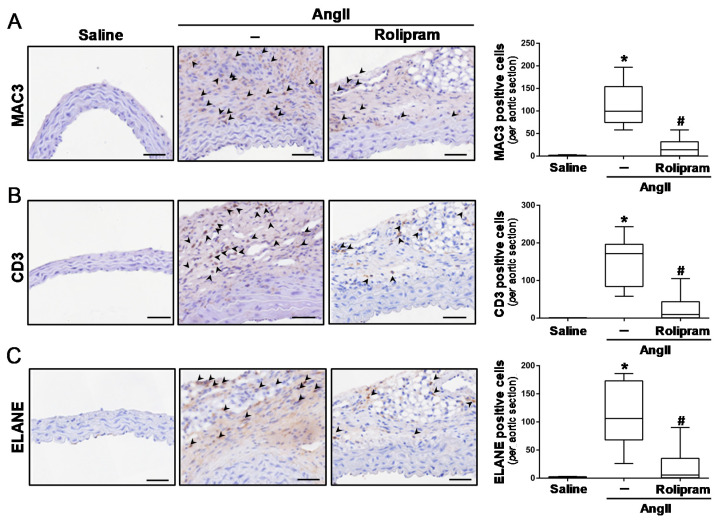
Inflammatory cell infiltration in the abdominal aorta from AngII-infused ApoE^−/−^ mice is attenuated by rolipram. ApoE^−/−^ mice were infused with saline solution or AngII (1000 ng/kg/min) for 28 days. The mice challenged with AngII were treated or not with rolipram (3 mg/kg/day). (**A**–**C**) Representative images corresponding to macrophage ((**A**) MAC3), lymphocyte ((**B**) CD3) and neutrophil ((**C**) neutrophil elastase, ELANE) infiltration, analysed by immunohistochemistry. The arrowheads indicate positive cells (Bars: 50 µm). The boxplots on the right show the quantification of the positive cells for Mac-3, CD3, and the neutrophil elastase per aortic section. The box extends from the 25th to the 75th percentile, and the median is indicated by a horizontal line. The whiskers represent the maximum and minimum values (saline-infused mice, *n* = 5; AngII-challenged mice, *n* = 8; AngII-challenged mice treated with rolipram, *n* = 6). In (**A**–**C**), *p* < 0.05: * vs. saline-infused mice; # vs. AngII-challenged mice.

**Figure 5:**
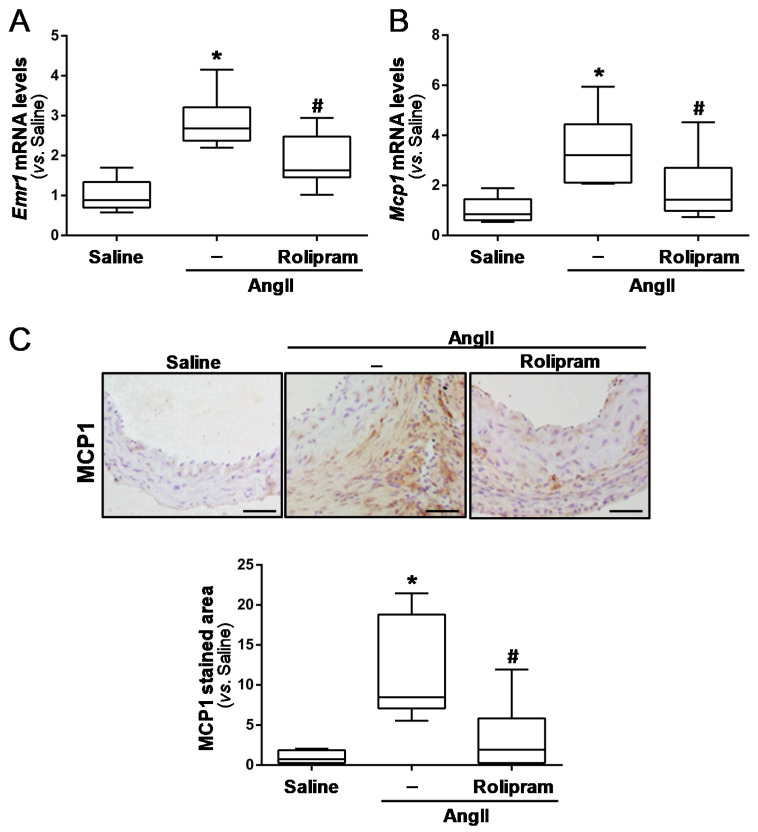
Rolipram limits the inflammatory response triggered by AngII in ApoE^−/−^ mice. ApoE^−/−^ mice were infused with saline solution or AngII (1000 ng/kg/min) for 28 days. Mice challenged with AngII were treated or not with rolipram (3 mg/kg/day). (**A**,**B**) The Emr1 (**A**) and Mcp1 (**B**) mRNA levels were analysed by real-time PCR (saline-infused mice *n* = 8; AngII-challenged mice, *n* = 12; AngII-challenged mice treated with rolipram, *n* = 9). (**C**) The immunohistochemical analysis of MCP1 expression in the abdominal aortas from each experimental group. Representative images are shown (Bar: 50 µm; saline-infused mice *n* = 5; AngII-challenged mice, *n* = 8; AngII-challenged mice treated with rolipram, *n* = 6). The data are presented as box and whisker plots. The box extends from the 25th to the 75th percentile, and the median is indicated by a horizontal line. The whiskers represent the maximum and minimum values. In (**A**–**C**), *p* < 0.05: * vs. saline-infused mice; # vs. AngII-challenged mice.

**Figure 6:**
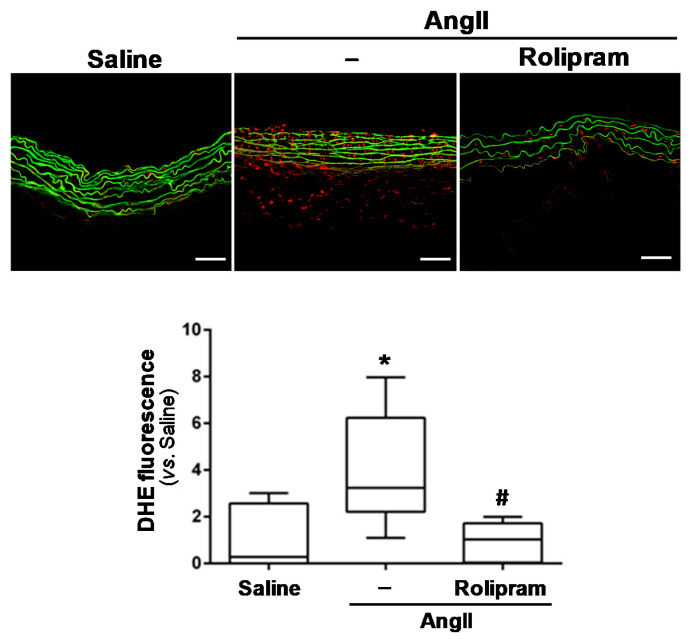
Rolipram reduces the increased production of vascular superoxide anions induced by AngII in ApoE^−/−^ mice. The ApoE^−/−^ mice were infused with saline solution or AngII (1000 ng/kg/min) for 28 days. The mice challenged with AngII were treated or not with rolipram (3 mg/kg/day). Their vascular superoxide anion production was assessed by DHE staining in the abdominal aorta sections of each experimental group. Representative images of the DHE staining are shown (Bars: 50 µm). The data are presented as box and whisker plots. The box extends from the 25th to the 75th percentile, and median is indicated by a horizontal line. The whiskers represent the maximum and minimum values (saline-infused mice *n* = 6; AngII-challenged mice, *n* = 8; AngII-challenged mice treated with rolipram, *n* = 7). *p* < 0.01: * vs. saline-infused mice; # vs. AngII-challenged mice.

Rolipram exhibits immunomodulatory and anti-inflammatory properties [19,34,35]. Therefore, we aimed to determine whether this drug limits vascular inflammation and oxidative stress, which are characteristics of aneurysmal disease. The immunohistochemical analysis evidenced that rolipram markedly reduced the infiltration of inflammatory cells evoked by AngII, resulting in less accumulation of macrophages (MAC3+ cells; Figure 4A), lymphocytes (CD3+ cells; Figure 4B), and neutrophils (neutrophil elastase+ cells; Figure 4C) into the aortic wall. Accordingly, rolipram ameliorated the vascular expression of Emr-1 (a macrophage marker) (Figure 5A), as well as that of monocyte chemoattractant protein-1 (Mcp1/Ccl2) (Figure 5B,C). Interestingly, the increase in superoxide anion levels detected in the aorta from AngII-infused mice by DHE staining was totally suppressed by rolipram treatment (Figure 6).

### 3.4. Rolipram Attenuates the Destructive Vascular Remodelling Triggered by AngII, and Limits MMP Expression and Activity

**Figure 7:**
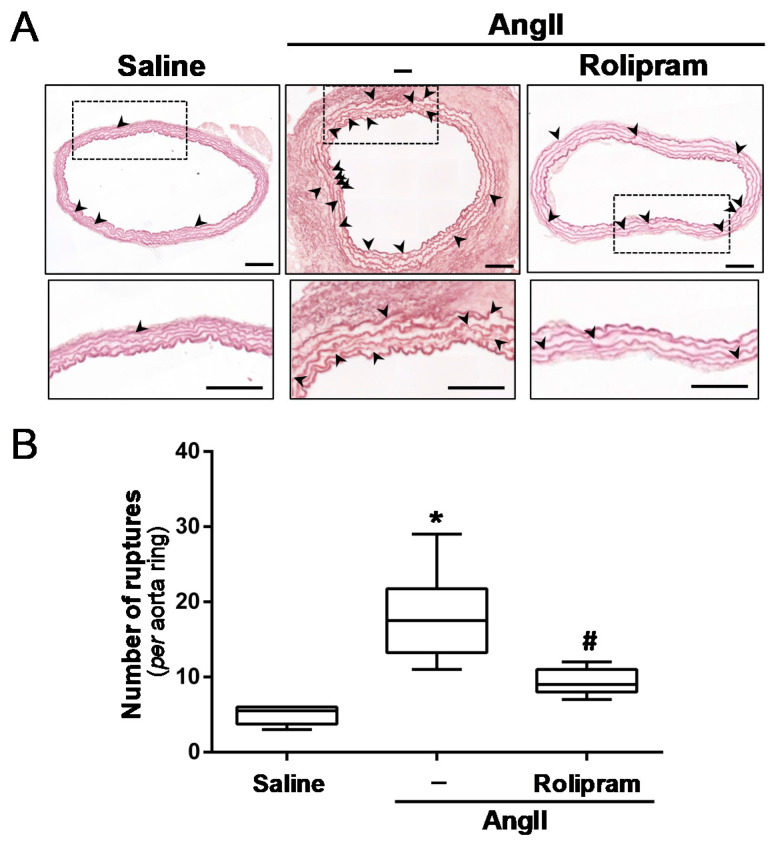
Rolipram attenuates the vascular remodelling induced by AngII in ApoE^−/−^ mice. ApoE^−/−^ mice were infused with saline solution or AngII (1000 ng/kg/min) for 28 days. The mice challenged with AngII were treated or not with rolipram (3 mg/kg/day). (**A**) Representative images showing the structure of the elastin fibres in the abdominal aortas after orcein staining. The arrowheads indicate elastin ruptures (Bars: 100 µm). (**B**) The boxplot shows the quantitative analysis of the number of ruptures in the elastin fibers per aortic section. The box extends from the 25th to the 75th percentile, and the median is indicated by a horizontal line. The whiskers represent the maximum and minimum values (saline-infused mice *n* = 6; AngII-challenged mice, *n* = 12; AngII-challenged mice treated with rolipram, *n* = 7). *p* < 0.05: * vs. saline-infused mice; # vs. AngII-challenged mice.

**Figure 8:**
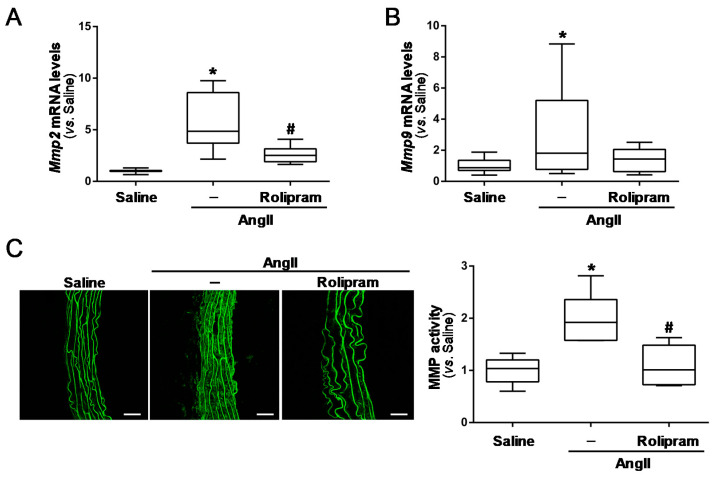
Rolipram attenuates the increase in MMP activity triggered by AngII in ApoE^−/−^ mice. ApoE^−/−^ mice were infused with saline solution or AngII (1000 ng/kg/min) for 28 days. The mice challenged with AngII were treated or not with rolipram (3 mg/kg/day). (**A**,**B**) The *Mmp2* (**A**) and *Mmp9* (**B**) mRNA levels were analysed by real-time PCR. The data are presented as box and whisker plots. The box extends from the 25th to the 75th percentile, and the median is indicated by a horizontal line. The whiskers represent the maximum and minimum values (saline-infused mice *n* = 8; AngII-challenged mice, *n* = 12; AngII-challenged mice treated with rolipram, *n* = 9). (**C**) The MMP activity analysed by in situ zymography in the abdominal aortas from each experimental group. Representive images are shown (Bars: 50 µm). The boxplot on the right shows the quantification of the MMP activity (saline-infused mice *n* = 5; AngII-challenged mice, *n* = 6; AngII-challenged mice treated with rolipram, *n* = 6). In A to C, *p* < 0.05: * vs. saline-infused mice; # vs. AngII-challenged mice.

We next sought to determine whether rolipram could impact on AngII-induced vascular remodelling. Importantly, rolipram preserved the elastin integrity, which was severely disrupted by AngII-infusion. Orcein staining shows that PDE4 inhibition reduced the high number of elastin fibre breaks detected in challenged mice (Figure 7A,B). In conjunction with the extensive disruption of elastic lamina evoked by AngII, a marked up-regulation of both the Mmp-2 and Mmp-9 mRNA levels was observed. Rolipram partially abrogated the AngII-mediated induction of Mmp-2 expression (Figure 8A), while a trend for the normalization of the Mmp-9 mRNA levels was detected, although the differences did not reach statistical significance (Figure 8B). Interestingly, in situ zymography in the aortic sections revealed that the PDE4B inhibitor abrogated the enhanced MMP activity induced by AngII (Figure 8C).

## 4. Discussion

There is currently an imperative need for the identification of effective pharmacological therapies which allow the stabilization or regression of established AAA; however, the insufficient comprehension of the complex pathophysiological mechanisms underlying this disease is a serious pitfall for this purpose. In the search for new pharmacological targets in this disease, we have reported, for the first time, that vascular PDE4B expression is upregulated in both human and experimental AAA. Furthermore, the pharmacological inhibition of PDE4 attenuates vascular inflammation and oxidative stress, preserving vascular integrity, thereby preventing aneurysm progression and reducing AAA incidence and severity.

The PDE4 family encompasses a group of cAMP-specific PDEs encoded by four different genes, PDE4A–D, with each of them generating long and short variants due to the use of alternate promoters or differential splicing [13]. Similarly to other PDEs, the PDE4 subtypes differ in their regulation, tissue-expression, subcellular distribution, and protein interaction. In fact, selective knockdown evidences that each subtype undertakes specific and non-redundant biological functions [36]. In particular, PDE4B, which is the predominant subtype expressed in inflammatory cells, critically influences immune and inflammatory responses, and constitutes an appealing target for the development of new therapeutic agents against a wide range of disorders, specifically, inflammatory and autoimmune pathologies [19,34]. However, despite the facts that AAA is a chronic inflammatory disease, and innate or adaptive immune responses have emerged as promising targets to limit human AAA [4], the potential of PDE4 enzymes as therapeutic targets for this disorder has not been previously addressed. As a first approach to test this, we analysed PDE4B expression in aneurysmal aorta from AngII-infused ApoE^−/−^ mice. The accumulating evidence supports the supposition that the overactivation of the renin–angiotensin system (RAS) plays a critical role in the onset and progression of human AAA [37]. Consistently, the infusion of the main RAS effector (AngII) into ApoE^−/−^ mice induces the formation of AAA which resemble those found in humans, characterized by exacerbated inflammation, oxidative stress, and ECM disorganization, as we and others have reported [24,26,27,31]. Interestingly, we showed that, while PDE4B was hardly detected in the vascular wall from the control animals (saline-infused ApoE^−/−^ mice), it was markedly increased in AAA from the AngII-challenged mice located in inflammatory cells. In agreement with this, PDE4B, undetectable in healthy human aorta, was also significantly upregulated in human AAA, exhibiting a strong immunostaining in the inflammatory cells. Instead, the PDE4D expression was similar in healthy and diseased aorta, either in humans or in the murine model. These data suggest that PDE4B could be involved in aneurysm pathogenesis, while PDE4D might have a minor role in this disease. Although aging is an important risk factor in the pathogenesis of AAA, it should be highlighted that, in our study, there were no significant differences in age between the donors and the AAA patients (*p* = 0.114; Mann Whitney U test). Therefore, it seems that we can rule out that differences in age account for the increased expression of PDE4B observed in the human aneurysmal samples. Regardless of whether or not age modulates PDE4B in the vasculature, therapeutic strategies impacting on cAMP levels have been proposed as novel approaches for age-related vascular diseases [38].

In view of the potential contribution of the up-regulation of PDE4B to AAA pathogenesis, we evaluated the impact of PDE4 inhibition on the formation of AAA induced by AngII. We found that the systemic administration of rolipram, the prototypical selective PDE4 inhibitor, to AngII-challenged mice limited the growth of the aortic diameter, as assessed by ultrasonography, while reducing the incidence and severity of aneurysm. Preclinical studies analyzing the consequences of PDE4 blockade on AAA expansion had not been previously carried out, although the inhibition of PDE4 activity limits the progression of cerebral aneurysms [39], a disorder which shares some pathological characteristics with AAA, such as ECM proteolysis and inflammation. It has been previously reported that rolipram could impact on vascular contractility, inducing vascular relaxation in specific vascular beds [40], whereas the consequences of PDE4 inhibition on cardiac function and hypertrophy remains unclear [41,42]. Despite this potential vasodilator effect, in our experimental conditions, rolipram did not affect blood pressure levels, and the cardiac hypertrophy and systolic function in AngII-infused mice remained unchanged. Therefore, we ruled out that the beneficial action of rolipram on AAA could be attributable to blood pressure- or cardiac-dependent mechanisms.

The attenuation of the AngII-mediated increase of the aortic diameter observed in mice treated with rolipram results from combined anti-inflamatory and anti-oxidant effects, which provide an overall vasoprotection and th eimprovement of vascular remodelling. Rolipram significantly attenuated the oxidative stress induced by AngII, in agreement with previous studies showing antioxidant effects derived from PDE4 inhibition [21,22,23]. Remarkably, the rolipram-mediated inhibition of AngII-induced AAA was also associated with a potent anti-inflammatory effect, as it strongly reduced the local inflammatory infiltrate and the expression of inflammatory markers. It has been extensively reported that PDE4 inhibition exerts vasculoprotective actions against inflammation by inhibiting leukocyte–endothelial cell interactions, attenuating the platelet-mediated neutrophil recruitment at the sites of vascular injury, and improving the endothelial barrier integrity in different pathological settings [43,44,45,46]. Furthermore, the decrease of MMP expression and the consequent reduction of its activity, likely secondary to the decrease in MMP-producing inflammatory cells reported here, could account, at least in part, for the beneficial effect exerted by this drug on vascular integrity. Indeed, rolipram preserved the disruption of elastin fibres which is characteristic of the aneurysmal process. The impact of PDE4 inhibitors on MMP expression and/or activity has been previously reported, mostly in the context of pulmonary diseases, in which these drugs abolish the enhanced elastolytic activity driven by inflammatory stimulus [47] and attenuate MMP-2 and/or MMP-9 levels [48,49]. Taken together, therefore, our results and those described above suggest that the selective blockade of PDE4 activity could be a promising therapeutic strategy for a myriad of vascular pathologies with an inflammatory component, from AAA to atherosclerosis restenosis and venous thrombosis [43,44,45,46]. In fact, this could explain the low rate of cardiovascular events reported in patients with chronic obstructive pulmonary disease receiving the selective PDE4 inhibitor roflumilast [50].

Therefore, our study shows the preventive effect of rolipram treatment in the formation of experimental AAA. Further studies should be conducted in order to assess the potential of this drug to slow or arrest the growth of pre-established AAA, an experimental approach which more closely resembles the medical condition of AAA patients. Finally, it should be noted that rolipram shows high affinity for PDE4B; however, it exhibits side-effects, such as emesis, due to the inhibition of the PDE4D subtype. Owing to their potential anti-inflammatory activity, there is an active search of novel and selective PDE4B inhibitors [19,51] which avoid these PDE4D-related side-effects. Although several candidates have been developed, they have neither been tested in chronic preclinical studies (except for inhaled administration against respiratory diseases) nor incorporated into clinical practice. We cannot exclude that the inhibition of other PDE4 enzymes besides PDE4B might also account for the rolipram-mediated inhibition of aneurysm formation reported here, in particular PDE4D, which represents the major PDE4 variant in VSMC, and the second one in inflammatory cells behind PDE4B [18]. However, in view of the specific up-regulation of PDE4B in both human AAA and in a preclinical model, it could be hypothesized that selective PDE4B inhibitors might benefit AAA patients, and would constitute a promising therapeutic avenue for this disease.

In conclusion, inflammatory cell depletion or the blockade of cytokine production/activity has consistently shown beneficial effects preventing the formation of experimental aneurysms [52,53]. Our results provide an additional proof-of-concept supporting the targeting of inflammation/oxidative stress in general, and PDE4 in particular, as a therapeutic strategy for AAA. Novel specific PDE4B inhibitors would constitute a promising therapeutic avenue to fight against AAA.

## Data Availability

The data presented in this study are available on request from the corresponding author.

## Supplementary Materials

The following are available online at https://www.mdpi.com/2076-3921/10/3/460/s1, Figure S1: Original blots shown in Figure 1; Figure S2: PDE4D expression analysis in abdominal aortas from Ang II-infused ApoE^−/−^ mice; Figure S3: Blood pressure values in saline or AngII-infused ApoE^−/−^ mice treated or not with rolipram. Table S1: Patients’ and donors’ clinical features; Table S2: Systolic function in saline or AngII-infused ApoE^−/−^ mice treated or not with rolipram; Table S3: Bodyweight values.
