# Supplementary material for: Rolipram Prevents the Formation of Abdominal Aortic Aneurysm (AAA) in Mice: PDE4B as a Target in AAA
Source: Antioxidants (Basel). 2021 Mar 16;10(3):460. doi: 10.3390/antiox10030460 (PMC8000788; doi:10.3390/antiox10030460)
Supplement: Supplementary file 1 [file antioxidants-10-00460-s001.pdf]

Supplemental material

Rolipram prevents the formation of abdominal aortic aneurysm (AAA) in mice: PDE4B as a target in AAA

Saray Varona[1,2,3†], Lídia Puertas[2,3,4†], María Galán[2,3,4], Mar Orriols[1,2,3], Laia Cañes[2,3,4], Silvia Aguiló[3,4], Mercedes Camacho[2,3,4], Marc Sirvent[2,5], Vicente Andrés[2,6], José Martínez-González[1,2,3,*] and Cristina Rodríguez[2,3,4*]

[1]Departamento de Patología Experimental, Instituto de Investigaciones Biomédicas de Barcelona-Consejo Superior de Investigaciones Científicas (IIBB-CSIC), 08036, Barcelona, Spain.

[2]CIBER de Enfermedades Cardiovasculares, ISCIII, 28029, Madrid, Spain.

[3]Regulatory Mechanisms of Cardiovascular Remodelling Group, Instituto de Investigación Biomédica Sant Pau, 08041,Barcelona, Spain.

[4]Regulatory Mechanisms of Cardiovascular Remodelling Group, Institut de Recerca Hospital de la Santa Creu i Sant Pau (IRHSCSP), 08025, Barcelona, Spain.

[5]Angiology and Vascular Surgery Service, Hospital Universitari Germans Trias i Pujol, 08916, Badalona, Spain.

[6]Vascular Pathophysioly Area, Centro Nacional de Investigaciones Cardiovasculares Carlos III (CNIC), 28029, Madrid, Spain.

Figure S1

PDE4B

β- actin

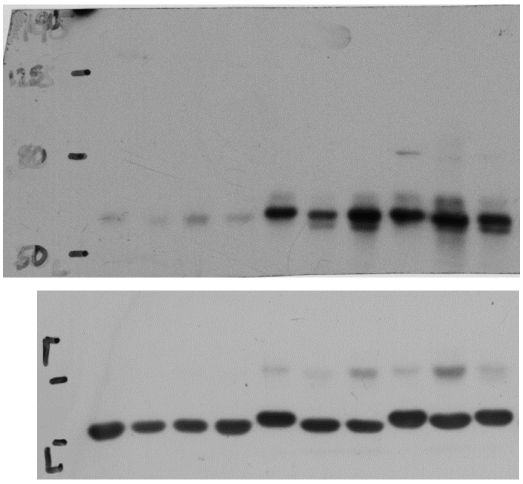

Figure S1. Original images corresponding to blots shown in Figure 1A.

Figure S2

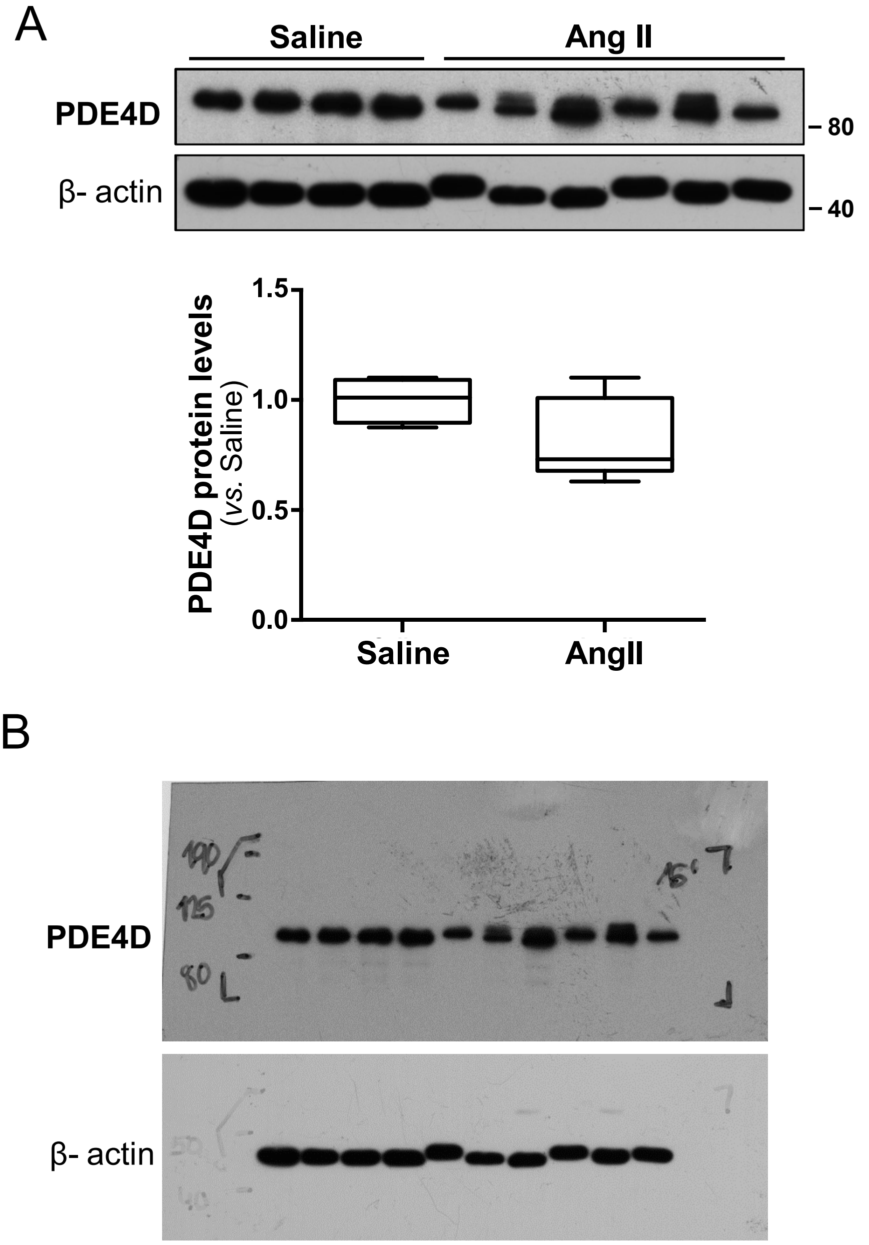

Figure S2. Ang II-infusion did not affect PDE4D expression in abdominal aortas from ApoE$^{-/-}$ mice. **A)** ApoE$^{-/-}$ mice were infused with AngII (1000 ng/kg/min) or saline solution for 28 days. PDE4D protein levels were assessed in abdominal aortas from these animals by Western-blot. Levels of β-actin are shown as a loading control. Protein size was estimated by the indicated position of molecular weight markers (in kDa). The Boxplot shows the quantification of PDE4D protein levels. Box extends from 25th to 75th percentile and the median is indicated by a horizontal line. Whiskers represent maximum and minimum values (Saline, n=4; Ang II n=6). **B)** Original images of blots shown in Figure S2A

Figure S3

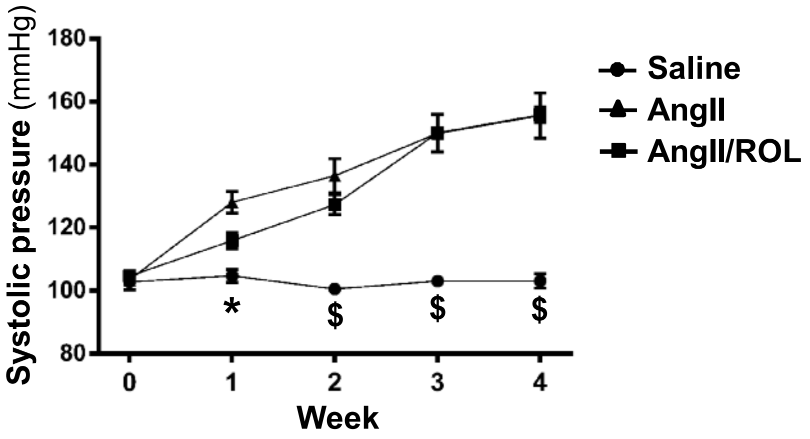

Figure S3. ApoE$^{-/-}$ mice were infused with saline solution (Sal) or AngII (1000 ng/kg/min) for 28 days. Mice challenged with AngII were treated or not with rolipram (ROL, 3 mg/kg/day). Blood pressure levels were assessed weekly in each experimental group. Data are mean±SEM (saline-infused mice, n=5; AngII-infused animals, n=11; AngII-infused mice treated with Rolipram, n=6). *P*<0.05: * *vs*. AngII-infused mice (at the same time); $ *vs.* AngII-infused mice treated or not with Rolipram.

Table S1. Patients and donors clinical features

Clinical parameters	Donors (*n* = 14)	AAA (*n* = 61)
Age (years ± SEM)	63.4 ± 16.8	70.7 ± 5.8
Males (%)	85.71	100
Smoking (%)*	57.1	83.6
Hypertension (%)	57.1	63.9
Diabetes (%)	35.7	8.2
Hyperlipidemia (%)	28.6	62.3
Ischemic cardiomyopathy (%)	0	14.8

* Current and ex-smokers. AAA, abdominal aortic aneurysm; SD, standard desviation.

Table S2. Systolic function parameters measured in saline or AngII-infused ApoE $^{-/-}$ mice treated or not with rolipram.

	**Saline**		**AngII**		**AngII+Rolipram**	
	**Basal**	**4 weeks**	**Basal**	**4 weeks**	**Basal**	**4 weeks**
**AWTd** (mm)	0,86 ± 0,097	0,82 ± 0,051	0,87 ± 0,021	1,07 ± 0,062*	0,92 ± 0,128	1,10 ± 0,073*
**PWTd** (mm)	0,76 ± 0,016	0,71 ± 0,035	0,76 ± 0,015	1,01 ± 0,068$^{\#}$*	0,75 ± 0,039	1,07 ± 0,072$^{\#}$*
**IVSTd** (mm)	0,81 ± 0,071	0,78 ± 0,070	0,85 ± 0,070	1,14 ± 0,068$^{\#}$*	0,83 ± 0,080	1,08 ± 0,080$^{\#}$*
**LVEF** (%)	63,28 ± 3,88	54,19 ± 4,47	52,77 ± 4,41	67,64 ± 3,37$^{\#}$*	54,31 ± 1,28	74,95 ± 2,26$^{\#}$*
**LVFS** (%)	33,83 ± 3,06	28,47 ± 2,77	27,93 ± 2,66	40,16 ± 2,61$^{\#}$*	28,04 ± 1,08	40,58 ± 1,41$^{\#}$*
**LV mass** (mg)	86,33 ± 6,13	91,19 ± 6,92	96,24 ± 6,68	120,61 ± 6,30$^{\#}$*	90,40 ± 7,18	118,50 ± 8,52$^{\#}$*
**LVEDV** (µL)	54,94±7,68	56,16±1,95	69,65±4,00	62,25±2,47	69,29±3,05	53,49±4,83
**LVESV** (µL)	22,08±2,96	26,52±3,03	31,98±5,82	22,82±2,99	31,84±1,41	15,04±2,77
**LV stroke volume** (µL)	32,85 ± 5,39	27,93 ± 2,52	37,66 ± 2,90	39,43 ± 2,59	37,45 ± 2,10	38,45 ± 3,02
**HR** (bpm)	408 ± 12,73	418,7 ± 12,25	381 ± 9,69	381,3 ± 9,44	386 ± 21,67	396,3 ± 16,49
**CO** (mL/min)	13,50 ± 1,55	11,81 ± 1,39	12,62 ± 0,69	15,7 ± 0,99	14,85 ± 1,53	14,34 ± 1,49
**n**	6	6	6	6	4	4

AWTd: end-diastolic LV anterior wall thickness; PWTd: end-diastolic LV posterior wall thickness. IVSTd: end-diastolic interventricular septal thickness; LVEF: LV ejection fraction; LVFS: LV fraction shortening; LVEDV: LV end-diastolic volumen; LVESV: LV end-systolic volumen; HR: heart rate; CO: cardiac output. Results are expresses as mean ± SEM. $p<$ 0.05: * *vs*. Saline for 4w; # *vs*. the same experimental group under basal conditions.

Table S3. Body weight in saline- and AngII-infused mice treated or not with Rolipram

Week	**Saline**	**AngII**	**AngII + Rolipram**
**0**	26.92±1.37	25.44±0.44	27.37±0.62
**1**	27.66±0.81	27.43±0.49	28.56±0.46
**2**	27.38±0.87	27.93±0.51	27.78±0.39
**3**	27.61± 0.86	27.72±0.59	27.75±0.44
**4**	27.33± 0.92	27.32±0.58	27.43±0.65

Results (in g) are expressed as media ± SEM. Saline, n= 6; AngII, n= 11; AngII + Rolipram, n= 6
